# Classification of Diabetic Retinopathy Disease Levels by Extracting Spectral Features Using Wavelet CNN

**DOI:** 10.3390/diagnostics14111093

**Published:** 2024-05-24

**Authors:** Sumod Sundar, Sumathy Subramanian, Mufti Mahmud

**Affiliations:** 1 School of Computer Science and Engineering, VIT Vellore, Vellore 632 014, India; sumod.sundar2016@vitstudent.ac.in; 2 School of Computer Science Engineering and Information Systems, VIT Vellore, Vellore 632 014, India; 3 Department of Computer Science, Nottingham Trent University, Nottingham NG11 8NS, UK; 4 Computing and Informatics Research Centre, Nottingham Trent University, Nottingham NG11 8NS, UK; 5 Medical Technologies Innovation Facility, Nottingham Trent University, Nottingham NG11 8NS, UK

**Keywords:** diabetic retinopathy, Wavelet CNN, classification, convolutional neural network, spectral features, computer-aided diagnosis

## Abstract

Diabetic retinopathy (DR) arises from blood vessel damage and is a leading cause of blindness on a global scale. Clinical professionals rely on examining fundus images to diagnose the disease, but this process is frequently prone to errors and is tedious. The usage of computer-assisted techniques offers assistance to clinicians in detecting the severity levels of the disease. Experiments involving automated diagnosis employing convolutional neural networks (CNNs) have produced impressive outcomes in medical imaging. At the same time, retinal image grading for detecting DR severity levels has predominantly focused on spatial features. More spectral features must be explored for a more efficient performance of this task. Analysing spectral features plays a vital role in various tasks, including identifying specific objects or materials, anomaly detection, and differentiation between different classes or categories within an image. In this context, a model incorporating Wavelet CNN and Support Vector Machine has been introduced and assessed to classify clinically significant grades of DR from retinal fundus images. The experiments were conducted on the EyePACS dataset and the performance of the proposed model was evaluated on the following metrics: precision, recall, F1-score, accuracy, and AUC score. The results obtained demonstrate better performance compared to other state-of-the-art techniques.

## 1. Introduction

Diabetic retinopathy (DR) is a medical condition that arises as a consequence of Diabetes Mellitus (DM). DM happens due to micro- and macrovascular abnormalities, as well as impaired glucose metabolism, resulting in chronic disease [1]. DR represents a prevalent and significant complication of DM, ultimately leading to profound visual impairment due to structural alterations in the retina. The leading factor behind the clinical symptoms of diabetes is the rise in blood glucose levels over an extended duration, which can adversely affect the blood vessels in the retina. The primary indicators of DR consist of the presence of microaneurysms, exudates, haemorrhages, and vascular edema within the ocular blood vessels. This condition frequently results in visual impairment and blindness among individuals ranging from 20 to 74 years of age. According to research studies conducted by the International Diabetes Federation, it is estimated that approximately 425 million individuals are currently affected by DR. Furthermore, this number is projected to rise to about 693 million by 2045 [2]. Early detection of DR plays a crucial role in preserving the patient’s vision. Diagnosis of DR can be performed through either manual examination by an ophthalmologist or by utilising an automated system. With the advancements in Artificial Intelligence (AI) techniques, automated system development has been facilitated in many application areas including anomaly detection [3], brain signal analysis [4], neurodevelopmental disorder assessment and classification focusing on autism [5,6,7], neurological disorder detection and management [8], supporting the detection and management of the COVID-19 pandemic [9], cyber security and trust management [10,11,12,13], various disease diagnosis [14,15,16,17], smart healthcare service delivery [18,19], text and social media mining [20,21], understanding student engagement [22,23], etc. As can be seen in the literature, automated systems for early disease detection have been a major area of development. Using an automated system for detecting DR has numerous benefits over manual detection methods. By implementing an automated system for detecting DR, the effort of ophthalmologists can be reduced, minimising the chances of human error. Furthermore, an automated system has the potential to detect lesions and abnormalities more effectively and efficiently than manual methods, thereby enhancing overall detection accuracy.

DR grading is efficiently performed by extracting latent feature distributions, and the probabilistic features make the multiclass labelling more accurate [24]. In the works of [25], the aim was to extract features utilising a Variational Autoencoder (VAE) and subsequently identify the underlying topological correlations by employing Graph Convolutional Neural Networks (GCNN). The combination of semantic information derived from high-level features and spatial information extracted from low-level data led to identifying vessels with diverse shapes and scales [26]. A DeepDR Plus system proposed by Dai et. al used three models: metadata, fundus, and combined, to predict DR progression. The fundus model utilises ResNet-50 for feature extraction from fundus images, incorporating a soft-attention layer for feature selection. By combining fundus scores with metadata, prediction accuracy is increased [27]. To classify the Epiretinal Membrane (ERM) which appears as an abnormal semi-translucent film of fibrocellular tissue, Choi et. al designed a deep learning model. After GAN-based augmentation, ResNet50 and EfficientNetB0 were used as the backbone CNN models for classification. The model helped in synthesising realistic CFP images with the pathological features of ERM [28]. Gangwar and Ravi developed a novel CNN model with 10 layers to grade DR fundus images. The model focuses on detecting and classifying various elements of blood vessels associated with DR [29]. However, the model faced challenges acquiring deep characteristic learning, resulting in limited sensitivity, particularly in the mild and moderate DR classes. Gargeya and Leng [30] utilised deep residual learning, similar to ResNet, to train a customised convolutional network for DR detection in colour fundus images. They incorporated a convolutional visualisation layer to highlight heatmap-based regions. However, despite being computationally efficient, the network exhibited less sensitivity, specificity, and AUC values when tested on a small MESSIDOR dataset. Li et al. employed a multitask algorithm to generate a feature map from retinal images utilising the ResNet50 model [31]. Furthermore, attention modules were used to identify the correlation between two levels of severity in DR grades. Choi et al. proposed a deep learning- and machine learning-based model for multiclass classification of retinal diseases [32]. They employed a VGG-19 architecture with transfer learning based on random forest to carry out both ten-class and three-class classification tasks. The work examined the deep learning model’s effectiveness in 10-class classification, which was constrained by the scarcity of images in the STARE dataset. Research has demonstrated that using transfer learning techniques for image classification tasks consistently leads to good results [33].

Automated techniques have been widely applied in retinal image processing for feature extraction. However, there is still a research gap in classifying DR grades and utilising the spectral features found in fundus images. The contributions of the proposed work are summarised as follows:

- A novel approach is introduced that combines Wavelet CNN to extract spectral features from retinal fundus images to perform DR disease classification across multiple grades.
- The extracted features from the Wavelet CNN are utilised as inputs for an SVM classifier, enabling efficient and effective classification.

The paper is organised as follows: Section 1 elucidates the introduction and discusses prominent CNN-based techniques used in DR grading tasks. Section 2 covers wavelet-based techniques applied to retinal images and DR grading techniques experimented on the EyePACS dataset. The experimentation techniques are included in Section 3 and results are discussed in Section 4.

## 2. Related Works

### 2.1. Motivation

Wavelet CNN is a hybrid architecture that combines the strengths of wavelet transform and CNNs for signal and image processing tasks like signal denoising, feature extraction, and classification. A wavelet transform decomposes a signal into different frequency components using wavelets, which are localised wave-like functions. In Wavelet CNN, the input signal or image is passed through a wavelet transform, decomposing it into different sub-bands (frequency bands). Each sub-band is fed into a separate convolutional layer, allowing the network to learn features from different frequency components. These features are then combined using techniques like concatenation or fusion. The combined features are processed through additional convolutional and fully connected layers to perform the desired task, such as classification, denoising, or image reconstruction. By utilising both time and frequency information from wavelets, Wavelet CNNs can potentially extract more comprehensive and informative features compared to traditional CNNs. Wavelet CNNs have shown promising results in various tasks, including removing noise from signals while preserving the underlying information, and reducing image size while maintaining quality [34].

### 2.2. Wavelet-Based Techniques Applied to Retinal Images

Agboola et al. proposed wavelet image scattering as a method to obtain low-variance representations within a specific class using 2D channel representations of retinal fundus images. The objective was to detect glaucoma and demonstrate the effectiveness of wavelet image scattering in generating strong and streamlined representations of retinal fundus image data [35]. Abdel-Hamid proposed an adapted VGG16 network with transfer learning to classify retinal images into good or bad quality categories [36]. The modified network incorporates spatial and wavelet detail sub-bands as potential inputs. The experimental findings reveal that spatial and wavelet inputs consistently yield strong performance, regardless of whether the modified VGG16 network was used with or without transfer learning. Parashar and Agrawal proposed a new approach for classifying glaucoma stages, utilising the flexible analytic wavelet transform (FAWT) [37]. This method involves decomposing pre-processed images into sub-band images using FAWT. Entropies and fractal dimension-based features are extracted by applying ReliefF and sequential box-counting algorithms. Fisher’s linear discriminant analysis is employed to rank the extracted features, and the highest-ranked features are used for glaucoma stage classification using a least squares-Support Vector Machine classifier. A novel hyper-analytic wavelet (HW) phase activation function has been developed to grade retinopathy images. This function is explicitly designed to work with wavelet sub-bands. Unlike traditional approaches that discard negative coefficients representing crucial edge feature maps, the HW phase activation function transforms them. The imaginary part of the complex activation is generated through the hyper-analytic wavelet phase. Chandrasekaran and Loganathan have selected the hyper-parameter of an activation function to ensure effective activations by creating a monotonic magnitude spectrum [38]. They introduced a computer-aided diagnosis tool called GabROP, which combines discrete wavelet transform-based texture features with multiple deep learning (DL) models. The texture features are derived from various convolutional neural networks (CNNs) trained on different image sets. These texture features are subsequently merged with spatial deep features extracted from the original fundus images for each CNN. Attallah et al. applied the discrete cosine transform to concatenate the features from three different CNNs [39]. This fusion process effectively reduces the size of the resulting features. A two-dimensional tensor empirical wavelet transform is utilised to classify glaucoma stages automatically. The decomposed components obtained through the 2D-T-WET method prove valuable for extracting texture-based features. A Student *t*-test algorithm is employed to select and rank these features, identifying 12 robust features. These features are then used with the MC-LS-SVM classifier to achieve the highest classification accuracy, as reported by Parashar and Agrawal [40]. Rasti et al. presented a novel approach for automatically diagnosing abnormal macula in retinal optical coherence tomography images [41]. They utilised wavelet-based convolutional neural network (CNN) features and random forest classifiers. The proposed method generates CNN codes to represent B-scans by employing a wavelet-based CNN model designed explicitly for the spatial-frequency domain. Oliveira et al. presented a novel method that combines the multiscale analysis of the Stationary Wavelet Transform with a multiscale fully convolutional neural network [42]. This approach was specifically developed to tackle the challenge posed by the varying width and direction of the vessel structure in the retina. Li et al. proposed a technique for image enhancement by decomposing the grey retinal image using the Dual-Tree Complex Wavelet Transform (DTCWT) [43]. This decomposition yields high-pass and low-pass sub-bands. Specifically, the high-pass sub-bands are enhanced using a Contourlet-based method.

### 2.3. Doctor Grading Tasks Using EyePACS Dataset

To improve the model’s performance, the input image size was reduced from 3888 × 2951 to 786 × 512 [44]. Ensemble models, including Bagging, Boosting, and Stacking, were employed. Among these approaches, Stacking produced the best results. The proposed method combined five deep CNN models in an ensemble. The models used were ResNet50, Inceptionv3, Xception, Dense-121, and Dense-169. Categorical cross entropy was employed as the loss function, and the Nesterov-accelerated Adaptive Moment Estimation (Nesterov-Adam) was used as the optimiser. The experiment was conducted for 50 epochs, starting with an initial learning rate (α) of $10^{-2}$, then reduced by a factor of 0.1 to $10^{-5}$. Qummar et al. created a specialised CNN model based on Inception V3’s architecture [45]. This unique model follows a Siamese-like approach with weight-sharing layers. To ensure consistency, all images were resized to 299 × 299 pixels. In the pre-processing step, each pixel value in an image was adjusted by subtracting the weighted mean of the surrounding pixels and then adding a 50% greyscale value. Initially, the weights of the Inception V3 model, which was pretrained on the ImageNet dataset, were used. The optimiser employed was Adam. The training process took place on a server equipped with NVIDIA GeForce GTX1080TI graphics cards. Pao et al. resized the entire image to a size of 100 × 100 pixels [46]. From the retinal image, only the green component was extracted. The extracted green component was utilised to compute the entropy image, which was the main focus of their proposed method. A pre-processing step was performed using a technique called unsharp masking (UM) for image enhancement. A bi-channel CNN was utilised, which incorporates features from both the entropy images of the greyscale and the green component.

### 2.4. Research Gaps

Automated techniques have been widely applied in retinal image processing for feature extraction. However, there is still a research gap in classifying DR grades and utilising the spectral features found in fundus images. Spectral features can provide quantitative measurements of various retinal characteristics, such as vessel diameter, retinal thickness, and macular edema.

- Wavelet features are effective for texture analysis in retinal images. Without them, the ability to quantify and analyse different textures such as microaneurysms, exudates, or drusen is limited.
- Wavelet CNNs can effectively analyse retinal images at multiple scales, capturing both fine details and global features. Without them, the analysis is limited to a fixed resolution that leads to missing important structures at different scales.

## 3. Materials and Methods

### The Proposed Pipeline

**Figure 1:**
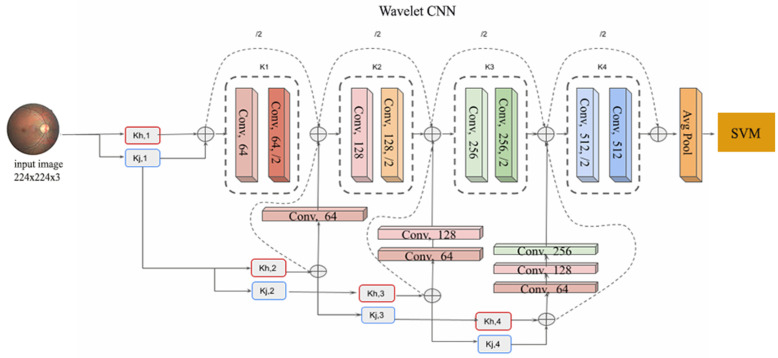
Proposed model for the classification of DR from fundus image.

The architecture of the proposed model is shown in Figure 1. The Wavelet CNN architecture utilises several techniques to process input images. Initially, convolution layers are used with 3 × 3 kernels and 1 × 1 padding. Convolutional kernels of size 3 × 3 with a stride of 2 and 1 × 1 padding are used to reduce the feature map size. Additionally, the input image undergoes multiresolution analysis, decomposing it into different images representing various frequency components. These decomposed images are then concatenated channel-wise. Projection shortcuts are implemented using 1 × 1 convolutions. The output of the convolution layers is vectorised through global average pooling. The Support Vector Machine (SVM) classifier categorises the feature vector from the previous layer into different grades. Low-pass and high-pass filters are indicated by $k_{l}$ and $k_{h}$, respectively. Conventional CNNs can be considered a constrained version of multiresolution analysis using kernel $k_{t}$ without a characteristic hierarchical decomposition. This limited analysis fails to fully capture the rich hierarchical structure of multiresolution analysis, which can enable more efficient and effective information processing. To compensate for the missing part caused by $k_{h,t}$, the model introduces an extra set of $k_{l,t}$. These new filters are employed to establish multiresolution analysis using wavelet transform, which is integrated into the neural network architecture. Performing multiresolution analysis necessitates carefully selecting filters, as arbitrary filters cannot fulfil this purpose. The choice of filters, including $k_{l,t}$ and $k_{h,t}$, plays a critical role in capturing the desired features and characteristics of the input signal or image at various scales or resolutions. This is due to the fact that filters with distinct frequency responses can extract diverse information from the input data and represent it at varying levels of intricacy. Hence, the choice of filters is crucial in accomplishing effective multiresolution analysis.

A multiresolution analysis accomplishes a hierarchical decomposition of $k_{l,t}$ into $k_{l,t+1}$ and $X_{h,t+1}y$ iteratively, applying the equation with varying values of $k_{l,t}$ and $k_{h,t}$ at each time step *t*, where ↓ is the standard downscaling operator.

(1) $$X_{l,t+1}=\left(X_{l,t}*k_{l,t}\right)↓2$$

(2) $$X_{h,t+1}=\left(X_{l,t}*k_{h,t}\right)↓2$$

In a multiresolution analysis, the term “level” refers to the number of applications, denoted as *t*. By reformulating, convolutional neural networks (CNNs) essentially disregard $X_{h,t}$ and utilise a single set of kernels, denoted as $k_{t}$.

(3) $$X_{l,t+1}=\left(X_{l,t}*k_{l}\right)↓2$$

In the context of wavelet transform, the filter $k_{h,t}$ is often known as the wavelet function, whereas the filter $k_{l,t}$ is referred to as the scaling function. The wavelet function captures the high-frequency components of the input signal, while the scaling function extracts the low-frequency components. These two filters comprise a comprehensive set of basic functions that have the capacity to represent the input signal or image across various scales or resolutions; while the experiments employ Haar wavelets, it is important to note that the model is not limited to Haar wavelets alone. This limitation also underscores the difficulty in training conventional CNNs to perform the same computations as Wavelet CNNs. This is because the CNN weights, represented by $k_{t}$, lack awareness of this critical constraint and instead rely solely on learning from the available datasets. Convolutional kernels of size 3 × 3 with 1 × 1 padding are used to maintain identical output dimensions as the input. This approach ensures that the output size matches the input size. To further reduce the feature map size, the architecture opts for convolution layers instead of pooling layers, incorporating an increased stride. Applying 1 × 1 padding to a layer with a stride of two reduces the output size to half of the input layer. This technique is an alternative to max pooling, as it preserves accuracy without any loss. Another commonality between the VGG-like architecture and image decomposition in multiresolution analysis is the progressive halving of the image size. Exploiting this shared characteristic, each level of decomposed images can be merged with feature maps from a specific layer that matches the size of those images. This approach performs efficient information fusion across different scales and resolutions, resulting in a more comprehensive and detailed representation of the input data. Incorporating dense connections in the model establishes a direct link between every level of decomposed images and all subsequent layers. This connection is established via channel-wise concatenation, ensuring a smooth information flow across the entire network. By establishing this connectivity, the model effectively harnesses information from all levels of decomposed images and integrates it with information from subsequent layers. This enables the model to effectively capture and utilise all relevant information for accurate predictions. Dense connections play a pivotal role in the network architecture that maintains an efficient and effective information flow. One approach to increasing input dimensions in a neural network is to employ projection shortcuts using 1 × 1 convolutional kernels. The model applies projection shortcuts in each shortcut path, as the feature map dimensions vary before and after the shortcut. This technique ensures that the network can effectively capture and propagate information across different layers, even when the feature map dimensions are inconsistent. The global average pooling is used instead of fully connected layers to prevent overfitting. This strategy effectively reduces the number of parameters within the network and enhances the model’s generalisation performance as a whole. Wavelet CNNs utilise global average pooling with an input size identical to that of the layer. This approach necessitates a fixed input image size for optimal performance. The model was exclusively trained in the experiments using images of size 224 × 224. Maintaining a consistent input image size ensures that the network’s architecture remains stable during training, allowing the model to be fine-tuned for accurate predictions, specifically on images of that size. Although this requirement may limit the model’s adaptability to handle images of different sizes, it can enhance its overall performance for the designated input size. To prepare the training images for our model, they were resized into 256 × 256 pixels. Subsequently, random cropping was applied to extract images of size 224 × 224 pixels and incorporate image flipping. This operation was performed to enhance the model’s ability to avoid overfitting by augmenting the diversity of the training data. By implementing random cropping and flipping, we effectively expanded the training dataset and exposed the model to various variations of the input images. This process aided in enhancing the model’s generalisation ability and the accuracy of its predictions on new images. To enhance the model’s robustness, batch normalisation was implemented across the network before the activation layers during the training phase. Batch normalisation is a prominent technique for mitigating the impact of covariate shifts, thereby improving the model’s stability and convergence during training. Instead of the conventional SGD optimiser, the Adam optimiser was used. This optimiser has advantages such as accelerated optimisation and convergence to superior solutions.

## 4. Classifiers

The extracted features from previous layers of Wavelet CNN are inputted into the classifier for disease grade classification. SVM, random forest, and XGBoost classifiers were used in the experiments.

### 4.1. Support Vector Machine

Support Vector Machine (SVM) is a powerful and versatile machine learning algorithm used for classification and regression tasks. SVMs are especially good when dealing with complex datasets and problems that involve finding decision boundaries between different classes. SVM aims to find the hyperplane that maximises the margin between the classes, known as the maximum margin hyperplane. The margin is defined as the distance between the hyperplane and the nearest data points from each class, which are called support vectors. This will help for robust generalisation and better handling of new unseen data. The fundamental principle behind SVM is to find the optimal hyperplane that maximally separates data points belonging to different classes [47]. This hyperplane is determined by a subset of data points called support vectors, which are the closest points to the decision boundary. It can handle datasets with a large number of features as it only requires a subset of support vectors for decision-making, reducing computational complexity. SVM incorporates a regularisation parameter (C) that controls the trade-off between maximising the margin and minimising classification errors. A small C value results in a wider margin but may allow some misclassifications, while a larger C value aims to classify all training examples correctly, possibly leading to a narrower margin. Furthermore, it has strong classification capabilities and moderate- to high-dimensional feature spaces.

### 4.2. XGBoost

XGBoost, which stands for “Extreme Gradient Boosting”, is a versatile and efficient machine learning algorithm known for its exceptional performance in predictive modelling tasks. XGBoost is based on the gradient boosting framework, which is a powerful ensemble learning technique and it is good in handling regression, classification, and feature scoring tasks [48]. It utilises a gradient descent algorithm to minimise the loss function. It builds predictive models by combining the outputs of multiple weak models (usually decision trees) to create a strong predictive model. In this approach, decision trees are used as predictors, with each tree sequentially improving predictions by reducing the error of the previous tree. XGBoost assigns weights to new trees based on their performance in the previous trees, and the overall prediction is obtained by summing the outputs from each predictor. Apart from regression and classification tasks, XGBoost also provides a feature importance score, which indicates how frequently each feature is used in the trees. This score can be mapped to threshold values to determine the optimal number of features and achieve maximum accuracy during training.

### 4.3. Random Forest

Random forest is based on the ensemble learning principle, where multiple decision trees are trained independently and then combined to make predictions used for regression and classification tasks. This ensemble approach helps mitigate the shortcomings of individual trees. It entails building numerous decision trees during the training phase, and the final prediction is made by selecting the class that appears most frequently across the individual trees [49]. Random forest introduces feature randomisation by selecting a random subset of features when making each split in the decision tree. This reduces the risk of trees fitting to noise and leads to better generalisation. Each tree in a random forest is trained on a bootstrapped (randomly resampled) subset of the training data. This diversity in data subsets reduces overfitting and enhances generalisation. By combining multiple trees and using techniques like bagging and feature randomisation, random forest is less prone to overfitting, making it a reliable choice for various datasets. In the training process, a multitude of decision trees are created, and their combined outputs determine the input’s class. In the context of random forests, each decision tree produces a discrete probability distribution representing the possible classes. The final prediction is determined by selecting the mode of these distributions. This methodology is particularly useful for classification tasks, where the objective is to assign inputs to one of several classes. Combining the outputs of multiple decision trees enhances the model’s accuracy and reliability, as each tree may capture unique aspects of the input data. Random forest can handle a variety of data types, including numeric and categorical features, and is less sensitive to outliers and missing values. The random forest has become increasingly popular in medical imaging applications for classification purposes [50,51].

### 4.4. Experimental Setup

The proposed model was trained by utilising the layers of Wavelet CNN architecture. Instead of relying on pretrained weights, the model was trained from scratch using fundus images as input. This process generated independent weights specific to the newly trained model. The features obtained from Wavelet CNN training were subsequently passed to a classifier. It is worth noting that the proposed model does not employ pretrained weights and, therefore, cannot be considered a transfer learning technique. Experimental results with different classifiers, namely SVM, random forest, and XGBoost, further validated this observation. The Keras API in the TensorFlow platform was used for streamlined model creation with eager execution. The training took place on the “standard-gpu” configuration, utilising the NVIDIA Tesla V100 GPU unit installed on the PowerEdge R740 server. The NVIDIA Tesla V100 is a top-tier GPU built on the Volta architecture, featuring 640 Tensor cores and 5120 CUDA cores, which ensures exceptional performance for Artificial Intelligence and high-performance computing (HPC) tasks.

### 4.5. Dataset Description

**Figure 2:**
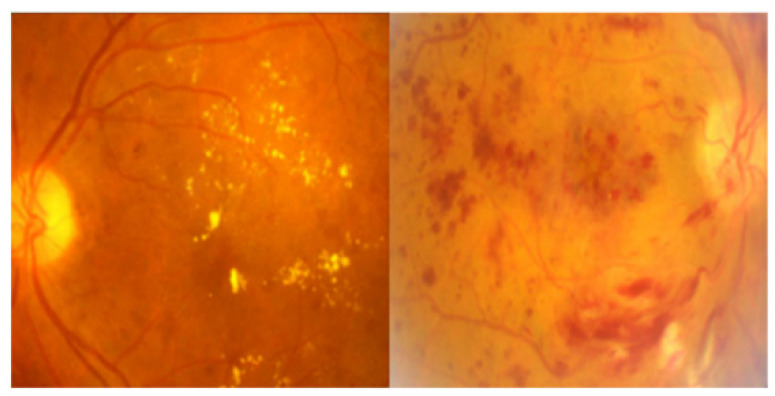
Representative images from the dataset—healthy eye (**left**) and eye with DR (**right**).

The EyePACS dataset is one of the largest publicly available datasets for DR. It contains retinal images acquired from diverse patient populations. Representative images of a healthy eye and an eye with DR are shown in Figure 2. The retinal fundus images in the dataset were captured using various imaging systems, including fundus cameras. Each retinal image in the dataset was given expert annotations or labels, indicating DR’s presence and severity. The dataset encompasses diverse patients, including different ethnicities, ages, and disease stages. This diversity enhances the generalisability of models trained on the dataset, making them applicable to various populations. Based on the International Clinical Diabetic Retinopathy (ICDR) severity scale, DR was further categorised into different grades that indicate the severity and progression of the disease. To extract hidden features, contrast enhancement was carried out to adjust the brightness and darkness of the image pixels. Fundus images exhibit a low contrast between the retinal background and blood vessels. To address the imbalance issue, augmentation techniques were applied to expand the size of both datasets. An iterative procedure was implemented during model training to generate data for each mini-batch. Techniques such as horizontal flip, width shift, height shift, fill mode, and zoom range were used to increase the number of images. Table 1 shows the DR severity grades and count of images before and after augmentation.

**Figure 3:**
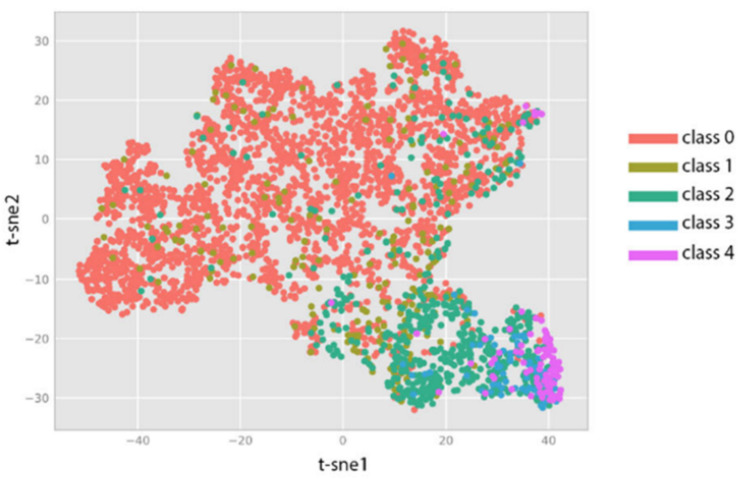
Latent vector space representation using t-SNE.

Figure 3 showcases the distribution of severity classes derived from the EyePACS dataset through t-SNE visualisation. It is evident that classes 0, 2, and 4 are clearly distinguishable in both spaces. However, there is a lack of clear separation between classes 0 and 1. In the scenario of classes 3 and 4, even though the separation is imperfect, it is still possible to distinguish a distinction in the spatial distribution of both classes in both spaces.

### 4.6. Metrics Used

The following metrics have been used to evaluate the proposed model’s performance. Precision: It measures the proportion of correctly predicted positive instances out of the total instances predicted as positive. It quantifies the model’s ability to avoid false positives.

(4) $$Precision=\frac{\left(True\;Positives\right)}{\left(True\;Positives+False\;Positives\right)}$$

Recall (Sensitivity): Recall, also known as sensitivity or true positive rate, measures the proportion of correctly predicted positive instances out of all actual positive instances. It quantifies the model’s ability to avoid false negatives.

(5) $$Recall=\frac{\left(True\;Positives\right)}{\left(True\;Positives+False\;Negatives\right)}$$

F1-score: The F1-score is the harmonic mean of precision and recall, providing a balanced measure between the two metrics. It is a single metric that combines precision and recall, offering an overall evaluation of a model’s performance.

(6) $$F1-score=2*\frac{\left(Precision*Recall\right)}{\left(Precision+Recall\right)}$$

Accuracy: It calculates the ratio of correctly classified instances to the total number of instances.

(7) $$Accuracy=\frac{\left(True\;Positives+True\;Negatives\right)}{\left(True\;Positives+True\;Negatives+False\;Positives+False\;Negatives\right)}$$

AUC Score: The AUC score measures the performance of a binary classification model by evaluating the area under the Receiver Operating Characteristic (ROC) curve. It quantifies the model’s ability to distinguish between positive and negative instances across various probability thresholds. AUC score ranges between 0 and 1, with higher values indicating better performance. An AUC score of 0.5 indicates a random classifier, while a score of 1 represents a perfect classifier.

## 5. Results and Discussion

### 5.1. Experimental Results

**Figure 4:**
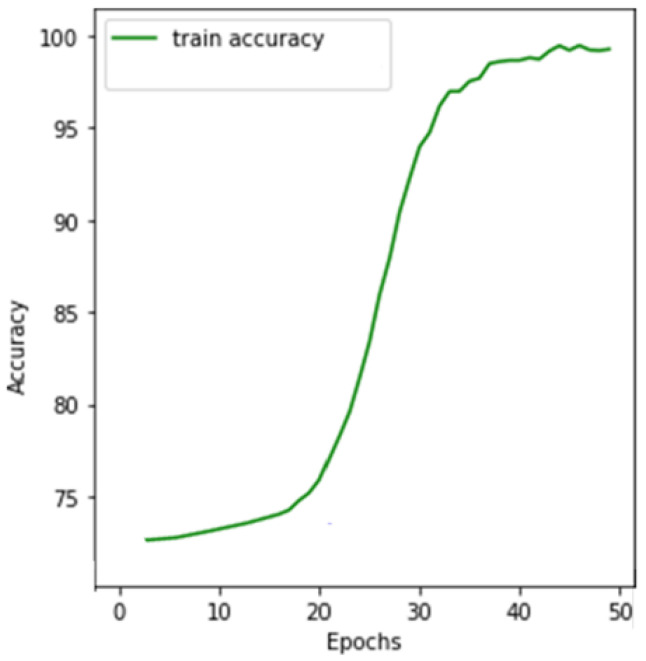
Training accuracy of Wavelet CNN.

The EyePACS dataset was then divided into 80%–20% for training and testing. The fundus images were trained with Wavelet CNN, and the architecture took 11,799,109 parameters to learn. The training converged with 50 iterations, which took 13.4 h to complete. The training accuracies of Wavelet CNN obtained over various iterations are shown in Figure 4. The training accuracy converged to 96.2% by the end of iterations with early stopping. Furthermore, the training loss converged to 0.35 by the end.

**Figure 5:**
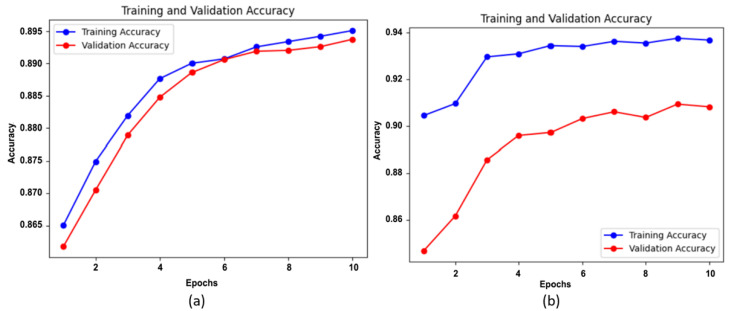
Training–validation accuracy of (**a**) XGBoost and (**b**) random forest.

**Figure 6:**
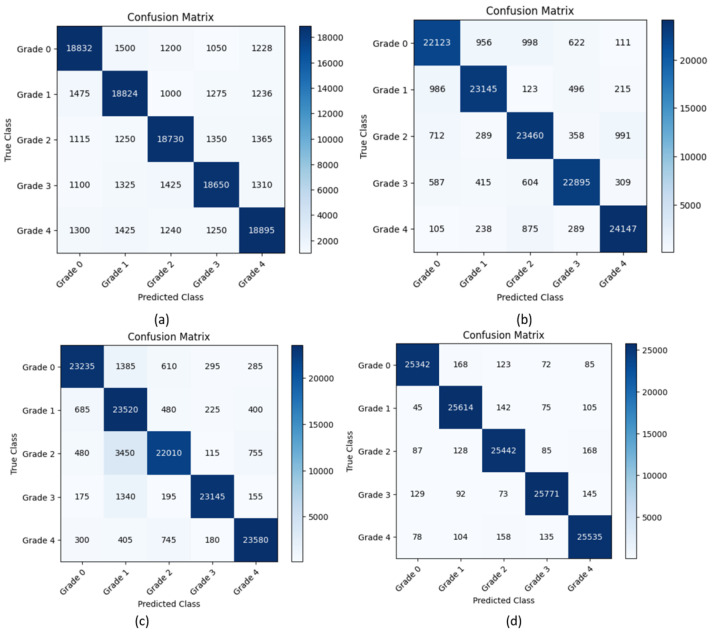
Confusion matrix: (**a**) Wavelet CNN, (**b**) XGBoost, (**c**) random forest, and (**d**) SVM.

**Figure 7:**
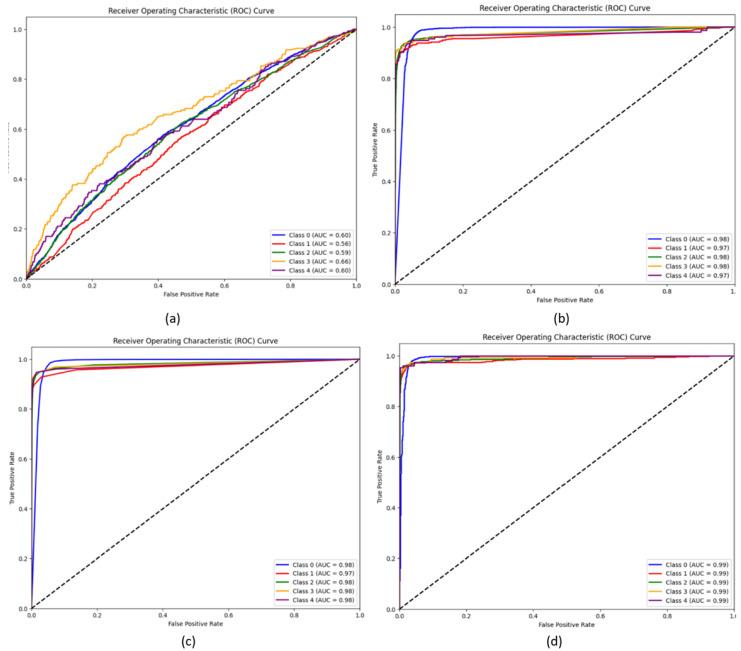
ROC curves: (**a**) Wavelet CNN, (**b**) XGBoost, (**c**) random forest, and (**d**) SVM.

The performance of the model, experimenting with various classifiers, was evaluated using the validation accuracy obtained. Initially, the performance of Wavelet CNN in feature extraction and classification was individually observed. Then, the features extracted from Wavelet CNN were fed into XGBoost, random forest and SVM classifiers. To prevent overfitting, the parameter n_estimators, was set as 10 for random forest and XGBoost classifiers. This parameter determines the number of boosting stages performed. Figure 5 display the training and validation accuracy independently achieved by XGBoost and random forest classifiers across various estimator epochs. The confusion matrices for XGBoost, random forest, and SVM are presented in Figure 6a–d. The ROC curves and AUC class scores for these classifiers can be seen in Figure 7a–d.

As shown in Table 2, the Wavelet CNN model demonstrates a decent performance in classifying DR into five grade classes. It achieves a precision of 0.857, indicating that it accurately identifies positive cases. The recall score of 0.849 suggests that the model effectively captures approximately 84.9% of the actual positive cases across the different grades of DR. The F1-score of 0.853 represents a balanced measure of precision and recall. However, the model’s accuracy is limited to 73% among five grade classes. Furthermore, the average AUC score of 0.602 (Figure 6a) suggests that the model’s ability to distinguish between positive and negative instances is relatively low. Incorporating XGBoost into the Wavelet CNN model shows notable improvements in the classification of DR into five grade classes. The combined model achieves a high precision of 0.9412, indicating an accurate identification of positive cases across different DR grades. The recall score of 0.8973 suggests that the model successfully captures approximately 89.7% of the actual positive cases across the different grades. The F1-score of 0.9186 signifies a harmonious balance between precision and recall. Moreover, the model’s accuracy significantly improves to 89.24%, indicating more correct classification. The high average AUC score of 0.976 (Figure 6b) indicates a strong ability to distinguish between positive and negative instances. The combination of the Wavelet CNN model with random forest yields a remarkable performance. The model achieves a precision of 0.940, implying the accurate identification of positive cases across different DR grades. With a recall score of 0.937, the model effectively captures about 93.7% of the true positive cases across diverse grades. It achieves a balanced trade-off between precision and recall, as reflected by the F1-score of 0.939. Furthermore, the model achieves a high accuracy score of 0.9095, indicating the correct classification of 90.95% of the cases across the five grade classes. The average AUC score of 0.978 (Figure 6c) shows good discrimination capability between the different DR grades. The model combining Wavelet CNN with SVM achieves a precision of 0.9831, recall of 0.9822, F1-score of 0.9831, accuracy of 0.9895, and AUC score of 0.99. The precision value shows a high proportion of true positives compared to false positives. The recall value indicates that the model captures almost all instances of the positive grade class. The F1-score of 0.9831 represents an excellent balance between precision and recall. The average accuracy of 0.9895 (Figure 6d) indicates a high rate of correct predictions. An AUC score of 0.99 indicates a strong ability to distinguish between positive and negative instances.

### 5.2. Comparison with Pretrained CNN Models

As shown in Table 3, the efficiency of the proposed Wavelet CNN with SVM is evaluated with pretrained models in the EyePACS dataset for DR classification. Qummar et al. [45] combined the predictions of five CNNs and achieved an accuracy of 0.857. However, its sensitivity (ability to detect true positives) and specificity (ability to detect true negatives) are relatively low (around 0.58), suggesting a potential issue with correctly classifying both positive and negative cases. The Inception-v3 model used by Zeng et al. [44] performed better than the ensemble in terms of specificity (0.9510), indicating its ability to identify true negatives. However, its accuracy (0.7070) and sensitivity (0.8282) are lower, suggesting room for improvement in overall classification performance. The pretrained ResNet model used by Wan et al. [52] achieved a very high accuracy (97.43) and reasonable specificity (95.68). However, its sensitivity (86.47) indicates ResNet’s difficulty in correctly identifying some true positive cases.

### 5.3. Comparison with Other Models Using EyePACS Dataset

**Figure 8:**
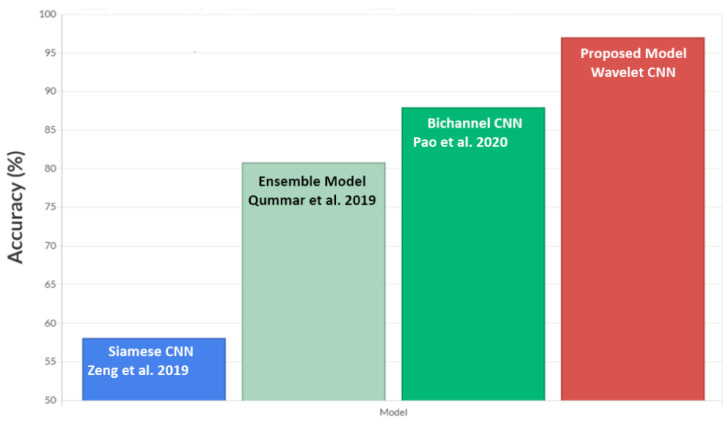
Accuracy comparison of the proposed model with other models using the EyePACS dataset [44,45,46].

The performance of the proposed model was evaluated by comparing it with other models previously used in the literature [44,45,46] for grading tasks of DR using the EyePACS dataset. The accuracy score achieved from the proposed model’s experimentation on the EyePACS dataset was compared with the results in the literature, as shown in Figure 8.

### 5.4. Discussion

Wavelet CNN efficiently extracts local features through wavelet decomposition, potentially capturing key characteristics of DR like microaneurysms and exudates. It is less computationally expensive than other deep learning models, especially for smaller datasets. SVM exhibited good interpretability, allowing the understanding of the features used for classification. By estimating the precision, recall, F1-score, accuracy, and average AUC scores from the ROC curves of various classifiers, Wavelet CNN, along with SVM, exhibited the best results. The results indicate that SVM outperforms XGBoost and random forest classifiers. The following observations have been inferred based on the results obtained:

- SVM can effectively learn complex decision boundaries to transform the data into a higher-dimensional space. This allows SVM to capture intricate relationships and patterns that might be missed by random forest and XGBoost.
- SVM is less sensitive to outliers compared to ensemble methods like random forest and XGBoost. SVM focuses on maximising the margin between different classes, which makes it more robust to noisy and outlier data points. On the other hand, decision trees in random forest and XGBoost can be influenced by outliers and may create biased splits.
- The hyperparameters of SVM, such as C, which defines the penalty for misclassification, and Gamma, which defines the influence of a single training example, reduce the risk of overfitting or underfitting.

Additionally, it was observed that the fully connected layer of the Wavelet CNN architecture was inadequate for classification purposes; therefore, classifiers such as SVM were necessary to effectively utilise the spatial features and achieve good classification results with a significant amount of data. The results in Table 2 indicate that SVM outperformed the other classifiers.

While comparing the proposed model with pretrained CNN models, it was found that wavelet transforms showed the ability to extract features at different scales and frequencies. This allows the Wavelet CNN to potentially capture both the detailed blood vessel abnormalities and larger structural patterns crucial for DR classification. The margin generated by SVM seems to be effective for DR classification by using the features extracted by the Wavelet CNN to separate healthy and DR-affected images.

When compared with other models that used the EyePACS dataset, a performance improvement of 10.44% was obtained in terms of accuracy score. The Siamese networks used in reference [44] require a substantial amount of data as they operate on pairs of classes. Additionally, these networks are highly sensitive to variations in the input. Complex relationships between features were not captured as effectively as with the WaveletCNN model. The Siameese model is potentially less robust as it may struggle with variations in image quality or diverse datasets compared to WaveletCNN. The ensemble model attempts to utilise and combine the features generated by different models [45]. The bi-channel model discussed in [46] necessitates a more significant number of trainable parameters, which leads to a more complex model. Moreover, it struggles to effectively extract neighbourhood information. Designing and training ensembles and bi-channel models is more complex than individual models, while Wavelet CNN + SVM offers interpretability and efficiency in DR classification, the training and inference of ensembles can be computationally expensive.

## 6. Conclusions

The need for a computer-aided diagnosis technique has increased for the early analysis of DR. Spectral features in an image refer to the characteristics or properties of different wavelengths or spectral bands present in the image which provide information about the intensity or colour distribution of the image at various wavelengths. Analysing the spectral features can help in tasks such as identifying specific objects or materials, detecting anomalies, or distinguishing between different classes or categories within an image. This work presents a model using Wavelet CNN and Support Vector Machine, which is evaluated to classify clinically significant grades of DR from retinal fundus images. The classification performance of SVM is compared against random forest and XGBoost algorithms using wavelet features. The proposed model achieved exceptional results, with an AUC of 0.99 and an accuracy of 98.95% when evaluated on the EyePACS dataset. The utilisation of wavelet features extracted from fundus images holds promise for enhancing the accurate grading of DR in real-time scenarios. This approach extracted more differentiable properties of the fundus image by capturing both the spatial and frequency patterns of retinal lesion structures. Wavelet CNNs have demonstrated superior or similar accuracies while utilising significantly fewer trainable parameters compared to traditional CNNs and pretrained CNN architectures. The experimental results demonstrate the efficient and accurate classification of the disease, ensuring its potential as a promising tool for clinical diagnosis and decision-making.

## 7. Future Directions

Standard Wavelet CNNs decompose an image into low- and high-frequency components using Discrete Wavelet Transform (DWT). Low frequency captures global structures, while high frequency captures fine details and noise. These networks primarily focus on feature extraction and classification, not directly visualising where the model focuses its attention. Wavelet CNNs do not directly generate attention maps like Grad-CAM. However, some recent advancements and variations make them achieve similar functionality. The Wavelet-Attention CNN (WA-CNN) integrates an attention mechanism into the Wavelet CNN architecture. DWT is used as before, separating frequencies. A Wavelet-Attention block is applied to the high-frequency component, assigning different attention weights to different details based on their importance. This effectively creates an attention map focused on the high-frequency, detail-rich areas. For future experiments, these strategies could further enhance performance.

## Figures and Tables

**Table 1:** EyePACS dataset—description.

Grade	Abnormality	No. of Images before Augmentation	No. of Images after Augmentation
Grade 0	No apparent retinopathy	25,810	25,810
Grade 1	Mild non-proliferative diabetic retinopathy (NPDR)	2443	25,810
Grade 2	Moderate NPDR	5292	25,810
Grade 3	Severe NPDR	873	25,810
Grade 4	Proliferative diabetic retinopathy (PDR)	708	25,810

**Table 2:** Performance evaluation of experiments with classifiers.

	Precision	Recall	F1-score	Accuracy	AUC Score
Wavelet CNN	0.857	0.849	0.853	0.73	0.602
Wavelet CNN + XGBoost	0.9412	0.8973	0.9186	0.8924	0.976
Wavelet CNN + Random Forest	0.940	0.937	0.939	0.9095	0.978
**WaveletCNN + SVM**	**0.9831**	**0.9822**	**0.9831**	**0.9895**	**0.99**

**Table 3:** Comparison with pretrained CNN models.

	Precision	Recall	Accuracy
Ensemble of 5 CNNs [45]	0.857	0.5810	0.5805
Inception-v3 [44]	0.7070	0.8282	0.9510
ResNet [52]	97.43	86.47	95.68
**Wavelet CNN + SVM**	**0.9831**	**0.9822**	**0.9895**

## Data Availability

This study used a public dataset which has been cited within the article. The dataset can be obtained from the lead authors, subject to prior institutional data-sharing agreements.
